# Is limited aortic resection more justified in elderly patients with type A acute aortic dissection?-insights from single center experience

**DOI:** 10.1186/s13019-020-01234-8

**Published:** 2020-07-23

**Authors:** Wei Qin, Cunhua Su, Liangpeng Li, Michael. Carmichael, Fuhua Huang, Xin Chen

**Affiliations:** Department of Thoracic and Cardiovascular Surgery, Nanjing First Hospital, Nanjing Medical University, 68# Changle Road, Nanjing, Jiangsu 210006 P.R. China

**Keywords:** Aortic dissection, Elderly, Aortic surgery, Outcomes

## Abstract

**Background:**

This study compared limited aortic repair (ascending, and /or hemi-arch replacement) versus extended-arch repair (ascending, arch and proximal descending aortic replacement) used for patients aged 65 or older, who had type A acute aortic dissection (AAD), analyzing the influence of the extent of aortic repair on outcomes.

**Methods:**

From January, 2001 to December, 2015, 103 patients aged 65 or older underwent operation due to type A AAD in Nanjing First Hospital. The cohort was divided into two subgroups according to the surgical approaches, including limited aortic replacement (LAR, *n* = 41) and total arch replacement + stent elephant trunk implantation (TAR+SET, *n* = 62).

**Results:**

There was no significant difference in gender, age, hypertension, diabetes, smoking, PCI history, atrial fibrillation, pericardial effusion, aortic valve insufficiency (≥ moderate), shock situation before operation, and Euro-score II between the two groups except limb malperfusion and tear location. The cross-clamp time, CPB time, intubation time, ICU stay time and hospital time were all significantly less in the LAR group than in the TAR+SET group. A total of 89 patients were discharged home successfully after operation, with a difference of hospital mortality (*P* = 0.04). The overall survival rates at 5-year follow-up were 82.5 ± 6.0% in LAR group and 75.2 ± 5.6% in TAR+SET group, but with no difference (*p* = 0.151). The freedom from adverse aortic events at 5-year was 84.3 ± 6.5% in LAR group versus 97.9 ± 2.1% in TAR+SET group, with a statistical difference (*p* = 0.03).

**Conclusion:**

These findings support limited aortic resection is acceptable for elderly patients with type A AAD if surgical principles allow.

## Background

Type A acute aortic dissection (AAD) is a catastrophic event with high mortality and morbidity, and still remains a great challenge for cardiac surgeons [1, 2]. For these type A AAD patients, almost all the literatures demonstrated the advantage of surgery over conservative therapy, particularly in the long-term follow-up [1–3]. Total aortic arch replacement and frozen elephant trunk implantation in the descending aorta is the most widely used treatment in China and has made great progress in treating type A AAD. Sun and his colleagues [4, 5] promoted this surgery with great success, reporting striking outcomes. He maintains that total aortic arch replacement and stent elephant trunk implantation in the descending aorta offers the advantage of a complete repair of aorta and lowers the likelihood of late re-intervention. However, patients with type A AAD in China are much younger than those in western countries [6, 7], total arch replacement and frozen elephant trunk implantation in western is not as widely used in elderly patients as it is in China. Therefore, a current topic of debate is about the extent of aortic repair in elderly patients who are characterized by weakness, poor surgical tolerance, and limited life expectancy. Should we perform limited aortic resection (LAR) which is technically easier but leaves a large part of the diseased aorta untreated, or should we give patients total aortic arch replacement and stent elephant trunk implantation in the descending aorta (TAR+SET) which can accomplish the advantage of a complete repair of aorta and reduce the likelihood of late re-intervention? Therefore, the aim of the current study is to compare the short and mid-term results between these two procedures used in elderly patients.

## Methods

We reviewed our institutional single-centre database to identify all type A AAD patients who received operations in Nanjing First Hospital from January, 2001 to December, 2015. This study was approved by the ethical committee of Nanjing First Hospital (320–2018) and all patients provided their consent.

### Patients

Patients were examined by enhanced CT and transesophageal echocardiography (TEE) to confirm the diagnosis and assess the true/false lumen, bifurcations involvement, tear location, aortic diameter, and heart/valve function. A total of 510 consecutive patients with type A AAD underwent surgical repair in our hospital from January, 2001 to December, 2015. Among these patients, there were 107 patients who were equal to or greater than 65 years. Patients with known connective tissue disorders were absolutely excluded. Moreover, patients who had previous cardiac surgery (*n* = 1) and patients with chronic renal failure who needed dialysis (*n* = 3) were all excluded. Finally, 103 patients were enrolled in this study, divided into two groups: LAR included 41 cases and TAR+SET included 62 cases.

### Surgical procedures

After general anesthesia, the arterial blood pressures of both the upper and lower limbs were monitored. Median sternotomy was performed. Right axillary artery and right atrial cannulation was used for cardiopulmonary bypass (CPB) and selected cerebral perfusion (SCP). A vent catheter via the upper right pulmonary vein was inserted into the left atrium to decompress the left ventricle. Patients were cooled to a nasopharyngeal temperature of approximately 24 °C via CPB. During the cooling process, the brachiocephalic arteries were exposed, and the proximal aortic surgery (such as aortic valve repair or replacement, Bentall procedure, coronary artery bypass graft, etc) was undertaken. CPB was discontinued when the nasopharyngeal temperature reached 24 °C. The brain continued to be perfused at a rate of approximately 5 to 10 ml/kg·min through the right axillary artery cannulation. In case of hemodynamic instability or severe pericardial tamponade, the femoral artery and femoral vein were used for cannulation and initiation of CPB before sternotomy. If the right axillary artery was unsuitable for cannulation, the femoral artery was chosen for cannulation, and brachiocephalic arteries were perfused directly for cerebral perfusion during circulatory arrest. **Procedure for TAR + SET**: The aortic arch was opened after circulatory arrest, an optimal size of stent elephant trunk (a catheter sheath containing the surgical stent graft, Microport, CHINA) was inserted into the true lumen of descending aorta, then deployed. The stent elephant trunk, the main trunk of tetrafurcate graft and the aortic wall were carefully sutured together. The perfusion of the lower body was restored via one of the four branches of tetrafurcate graft. Then the left carotid artery was rebuilt, and the patient was re-warmed. The proximal aorta was anastomosed to the distal graft to restore the blood supply of the heart. Then heart started to re-beat. The other two brachiocephalic arteries were then reconstructed. **Procedure of LAR**: The aortic arch was opened under circulatory arrest and SCP. The ascending aorta and right hemi-arch were replaced. In case of no tear or only mild hematoma in aortic arch, we preserved the aortic arch and only replaced the ascending aorta under cross-clamp. The choice of LAR or TAR+SET was made individually depending on the tear locations and patients’ conditions.

### Definitions

Malperfusion syndromes were defined as having symptoms or signs attributable to disturbed blood flow to end-organ systems. Malperfusion syndromes were classified as acute myocardial infarction (AMI), cerebrovascular accident (CVA), and visceral or peripheral (limb) malperfusion. Radiographic or intra-operative evidence of dissection involving corresponding aortic branch vessels was required for the diagnosis of malperfusion syndrome. Cardiogenic shock was reported, if the preoperative systolic blood pressure was less than 90 mmHg, or the patient required intravenous use of inotropic agent. Adverse aortic events were defined as aorta rupture, or diameter of aortic dissection aneurysm ≥55 mm, or re-do aortic surgery due to aortic dissection aneurysm during follow-up.

### Follow-up

The standard follow-up protocol for these patients was as follows: performing enhanced CT at least once a year; having a telephone interview or outpatient interview at least once a year. All follow-up data were obtained from our institution.

### Statistical analysis

Categorical variables were expressed as percentages, and continuous variables were expressed as mean ± standard deviation with range. Statistical analysis was performed using Student’s t-test, if variances were not equal (tested by Leven’s test), Mann- Whitney-U-Test was performed. Chi-squared test (Fisher exact tests if *n* ≤ 5) was used for categorical variables. Survival analysis was performed according to the methods of Kaplan-Meier, and statistical differences were analyzed using the log-rank test. All statistical analyses were performed with SPSS 13.0 software. All *P*-values less than 0.05 were considered statistically significant.

## Results

### Preoperative data

The comparison of perioperative variables between the two groups were summarized in Table 1. The primary analyses revealed that there was no significant difference in gender, age, hypertension, diabetes, smoking, PCI history, atrial fibrillation, pericardial effusion, aortic valve insufficiency (≥ moderate), and shock situation prior to operation between the two groups. There were 4 and 15 patients who had malperfusion syndromes in LAR group and TAR+SET group, respectively. Among these, limb malperfusion in LAR group obviously differed from that in TAR+SET group (LAR group: 0 vs. TAR+SET group: 7/62, 11.3%, *p* = 0.04). There were no significant difference in the proximal and distal extent of aortic dissection, and the involvement of major aortic branching arteries between the two groups. No significant difference in Euro-score II could be found between the two groups (LAR group: 17.1 ± 5.5 vs. TAR+SET group: 16.7 ± 6.3, *p* = 0.733). The detailed data were shown in Table 1.

**Table 1:** Demographics and clinical characteristics of the two groups

Variable	LAR (***n*** = 41)	TAR + SET (***n*** = 62)	***P***
Male, n (%)	29(70.7)	42(67.7)	0.748
Age	70.7 ± 3.8	69.5 ± 3.2	0.143
Hypertension, n (%)	41(100)	62(100)	1
Diabetes, n (%)	11(26.8)	15(24.2)	0.763
Smoking, n (%)	17(41.5)	26(41.9)	0.962
Previous PCI, n (%)	2(4.9)	3(4.8)	1
Atrium fibrillation, n (%)	2(4.9)	3(4.8)	1
Pericardial effusion, n (%)	11(26.8)	19(30.6)	0.677
AI (≥ moderate), n (%)	12(29.3)	19(30.6)	0.881
Shock, n (%)	7 (17.1)	13(21.0)	0.625
Malperfusion syndromes			
AMI, n (%)	2(4.9)	2(3.2)	1
CVA, n (%)	2(4.9)	2(3.2)	1
Spinal cord malperfusion, n (%)	0	1(1.6)	1
Renal artery malperfusion, n (%)	0	3(4.8)	0.274
Limb malperfusion, n (%)	0	7(11.3)	0.04
Intramural hematoma, n (%)	5(12.2)	6(9.7)	0.686
Branch involvements			
Coronary artery, n (%)	8(19.5)	10(16.1)	0.658
Arch branches, n (%)	25(61.0)	36(58.1)	0.769
Visceral artery, n (%)	15(36.6)	18(29.0)	0.421
Renal artery, n (%)	18(43.9)	22(35.5)	0.391
Proximal extent of dissection			
Sinus (root), n (%)	15(36.6)	18(29.0)	0.421
STC or above, n (%)	26(63.4)	44(71.0)	0.421
Distal extent of dissection			
Descending thoracic, n (%)	5(12.2)	7(11.3)	0.843
Suprarenal, n (%)	4(9.8)	3(4.8)	0.332
Infrarenal, n (%)	6(14.6)	6(9.7)	0.443
Iliac or beyond, n (%)	26(63.4)	46(74.2)	0.243
Euro-SCORE II,%	17.1 ± 5.5	16.7 ± 6.3	0.733

Abbreviations: *AI* aortic valve insufficiency, *AMI* acute myocardial infarction, *CVA* cerebrovascular accident, *STC* sinus-tube conjunction

### Intraoperative results and postoperative situation

Ascending aorta tear was found in 30 and 31 patients in LAR group and TAR+SET group, respectively, with a statistical difference (LAR group: 30/41, 73.2% vs. TAR+SET group, 31/62, 50%, *p* = 0.019). Right axillary artery was the most common cannulation site, accounting for more than two thirds in each group with no difference between the two groups (*p* = 0.732). The CPB time, cross-clamp time and circulatory arrest time were significantly less in LAR group than that in TAR+SET group, contributable to different surgical procedures. It should be noted that 5 patients in LAR group only did the ascending aorta replacement without circulatory arrest. The analysis also indicated that intubation time, ICU stay time, and hospital stay time in LAR group were all less than that in TAR+SET group. Concomitant surgery in proximal aorta did not differ in the two groups. In TAR+SET group, 2 patients underwent ascending-femoral artery bypass and 1 did femoral-femoral artery bypass surgery concomitantly because lower limb malperfusion was still present after surgery. Eleven patients in TAR+SET group suffered from severe postoperative complications, including 3 new acute kidney injury (4.8%), 1 new stroke (1.6%), 1 new paraplegia (1.6%), 1 paraparesis (1.6%), 5 tracheotomy (8.1%), and 1 gastrointestinal bleeding (1.6%). But, it must be noticed that no patients with these complications were observed in LAR group (*p* < 0.001). Two deaths (4.9%) were observed after operation in LAR group, including one died of multiple organ dysfunction syndrome (MODS) postoperative day 4, one died of heart failure postoperative day 1. By contrast, 12 patients (19.4%) died in TAR+SET group after operation, 4 of whom were attributed to MODS, 2 were attributed to fatal cerebral infarction, 2 were attributed to severe lung infections, 2 were attributed to right heart failure, 1 was attributed to sudden rupture of the descending aorta, 1 was attributed to gastrointestinal bleeding. Therefore, there were a total of 14 deaths in this current study, with an overall hospital mortality rate 13.6%. Fisher’s exact test showed that there was a significant difference in hospital mortality between the two groups (*p* = 0.042). All detailed data can be seen in Table 2.

**Table 2:** Intraoperative results and postoperative situation in the two groups

Variable	LAR (***n*** = 41)	TAR + SET (***n*** = 62)	***P***
Arterial cannulation sites			
Right axillary artery, n (%)	31(75.6)	45(72.6)	0.732
Femoral artery, n (%)	5(12.2)	6(9.7)	0.686
Both, n (%)	5(12.2)	11(17.7)	0.447
Tear location			
Ascending, n (%)	30(73.2)	31(50.0)	0.019
Aortic arch, n (%)	3(7.3)	4(6.5)	1
Beyond arch, n (%)	3(7.3)	21(33.9)	0.002
No tear (hematoma), n (%)	5(12.2)	6(9.7)	0.686
CPB time, min	161.5 ± 17.2	179.5 ± 22.1	< 0.01
Cross-clamp time, min	93.8 ± 15.1	119.4 ± 20.5	< 0.01
Circulatory arrest time, min	18.7 ± 2.6^a^	21.3 ± 2.3	< 0.01
Concomitant surgery in proximal			
+ AVR, n	4	9	0.557
+ CABG, n	2	4	1
+ Bentall, n	2	3	1
+ no-coronary sinus replacement, n	3	3	0.680
+ ascending-femoral artery bypass, n	0	2	0.516
+ femoral-femoral artery bypass, n	0	1	1
Major postoperative complications, n (%)	0	11(17.7)^b^	< 0.01
Dialysis due to new acute kidney injury, n	0	3	–
New stroke, n	0	1	–
New paraparesis, n	0	1	–
New paraplegia, n	0	1	–
Tracheotomy for lung infection, n	0	5	–
Gastrointestinal bleeding, n	0	1	–
Intubation time, d	2.2 ± 1.3	4.6 ± 3.7	< 0.01
ICU stay time, d	4.7 ± 3.4	7.7 ± 5.8	< 0.01
Hospital stay time, d	17.5 ± 2.6	21.1 ± 4.8	< 0.01
Hospital mortality, n (%)	2(4.9)	12(19.4)	0.042

Abbreviations: *CPB* cardiopulmonary bypass, *AVR* aortic valve replacement, *CABG* coronary artery bypass graft, *Bentall* a name of procedure, *ICU* intensive care unit

Notes: ^a^Only 36 patients were included because three patients underwent the ascending aorta replacement under cross-clamp without circulatory arrest.

^b^One patient had dialysis due to new acute kidney injury and gastrointestinal bleeding simultaneously

### Survival rate

**Fig. 1:**
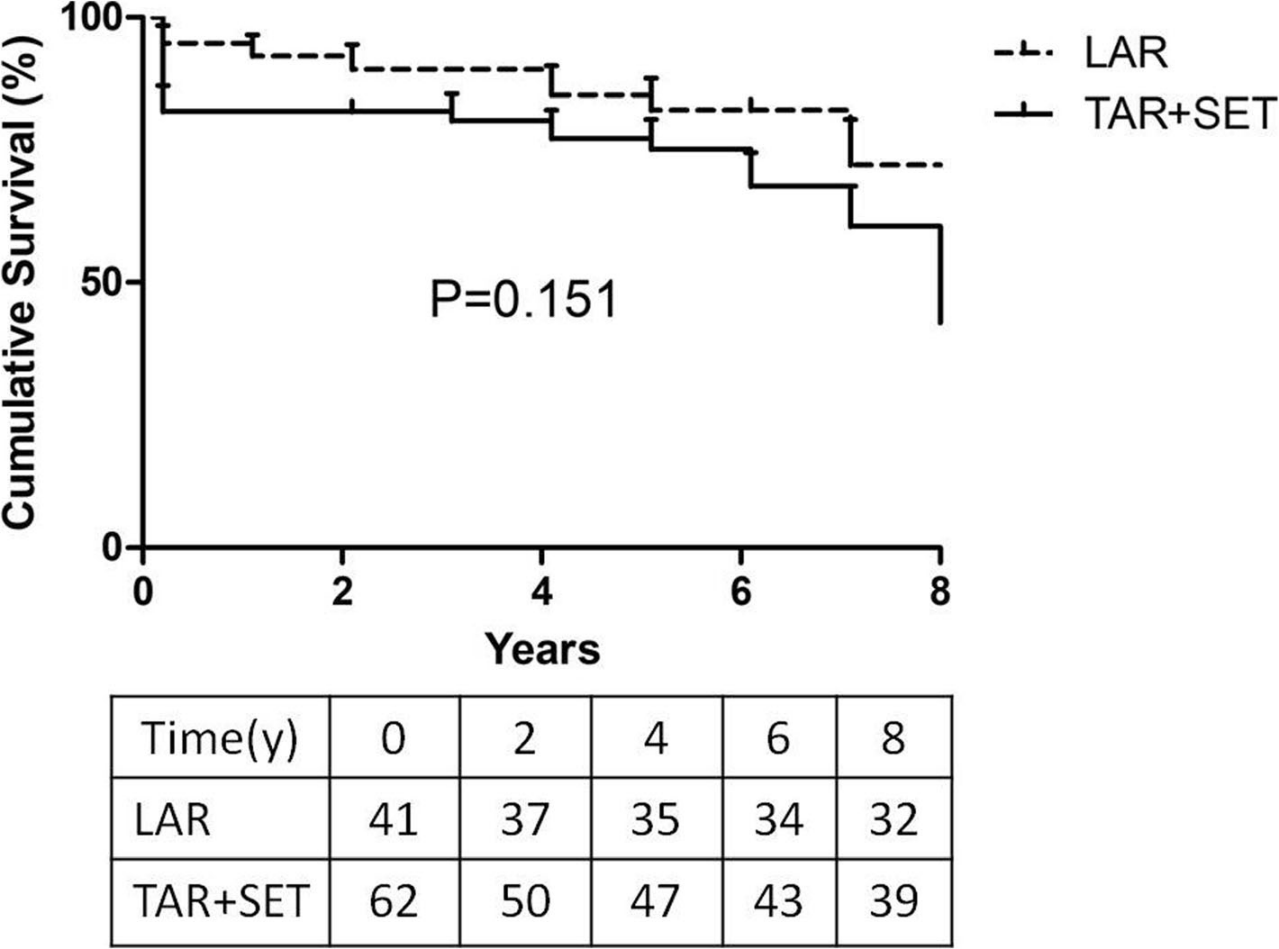
Comparison of survival between the two groups

Follow-up was successfully obtained in all discharged 89 patients with mean follow- up time (5.8 ± 1.6) years. Seven patients died in LAR group and 11 patients died in TAR+SET group during the follow-up. In LAR group, one died of re-do aortic surgery due to increasing aortic arch aneurysm. In comparison, no patient died of aortic dissection related reasons in TAR+SET group. Moreover, three patients died of unknown reasons in remote rural areas. The detailed causes of death in each group were described in Table 3. The Kaplan-Meier analysis estimated that 5-year survival rate was 82.5 ± 6.0% in LAR group versus 75.2 ± 5.6% in TAR+SET group; 7-year survival was 72.2 ± 8.6% in LAR group versus 60.6 ± 7.6% in TAR+SET group. We failed to find a significant difference in survival rate between the two groups (*p* = 0.151, see Fig. 1).

**Table 3:** Causes of death in the two groups

Causes of death	LAR (***n*** = 41)	TAR + SET (***n*** = 62)	***P***
Hospital death	2	12	0.042
Follow-up	7	11	0.637
Fatal CVA	2	2	–
Cancer	0	2	–
MI	0	1	–
Pneumonia	2	4	–
Dissection related	1	0	–
Traffic accident	0	1	–
Uncertain	2	1	–
5-year survival, %	82.5 ± 6.0	75.2 ± 5.6	0.151

Abbreviations: *CVA* cerebrovascular accident, *MI* myocardial infarction

### Adverse aortic events

**Fig. 2:**
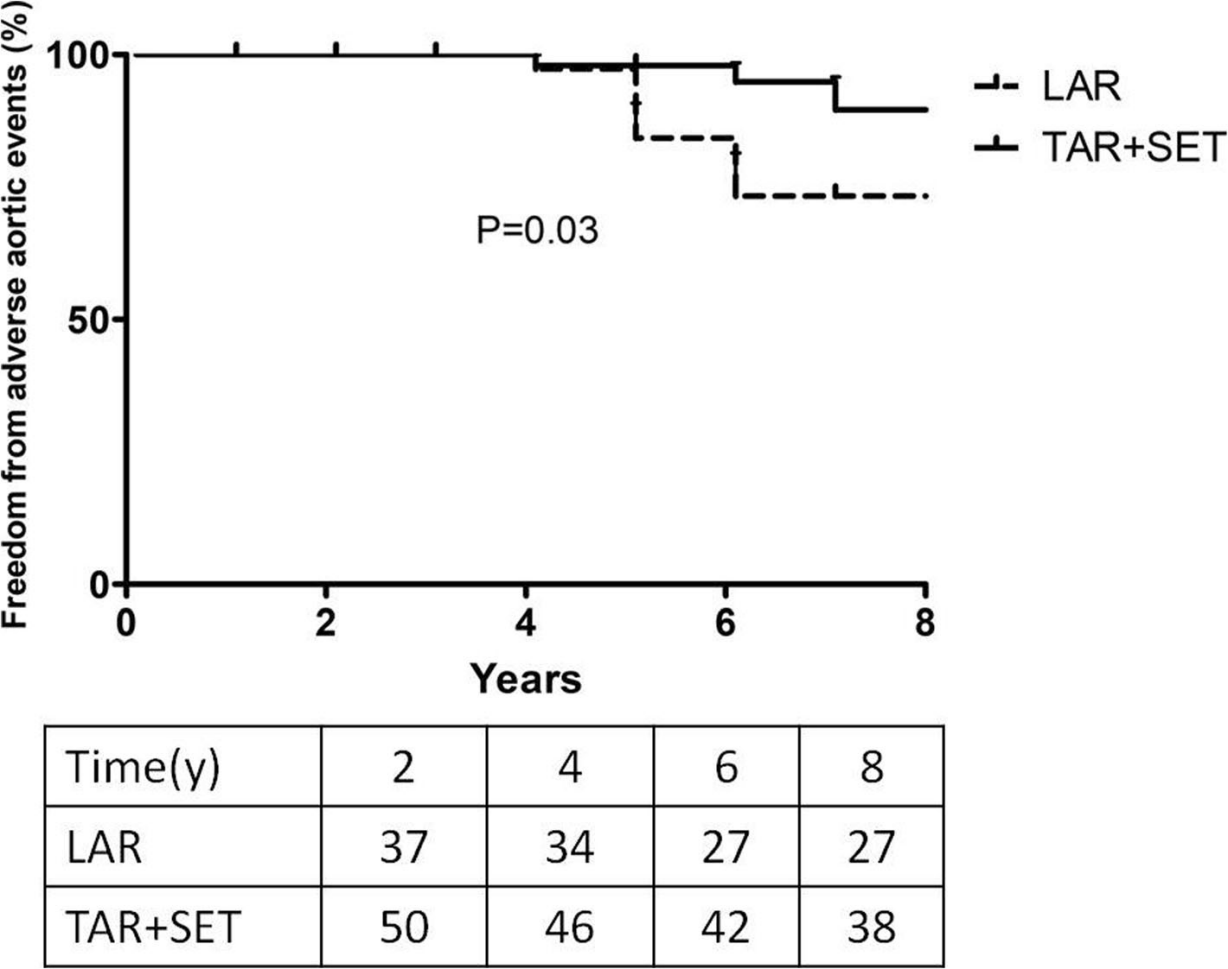
Freedom from adverse aortic events in the two groups

In LAR group, false lumen in aortic arch or proximal descending aorta was observed in 35 discharged patients (35/39, 89.7%). The increased diameter of proximal descending aorta was (19.5 ± 9.3) mm, with expansion occurring at (3.1 ± 1.6) mm/y for these 35 patients. Among them, aortic aneurysm progression increasing to ≥55 mm occurred in 8 patients (8/39, 20.5%) during follow-up. Three cases underwent re-do procedures because of the residual aortic aneurysm, one of whom died of MODS after re-do in ICU, the other two were discharged successfully after operations. The other 5 patients refused reoperations. By contrast in TAR+SET group, expansion of the true lumen and regression of the false lumen can be seen in aortic arch and proximal descending aorta for all the 50 discharged patients in follow-up CT. However, 3 patients had increasing abdominal aortic aneurysm with diameter ≥ 55 mm, but they refused re-interventions. The freedom from adverse aortic events at 5-year follow-up was (84.3 ± 6.5) % in LAR group versus (97.9 ± 2.1) % in TAR+SET group. Data analysis showed the freedom from adverse aortic events in LAR group differed from that in TAR+SET group (*p* = 0.03, Fig. 2).

## Discussion

Data from German Registry for Acute Aortic Dissection Type A (GERAADA) [3] showed that 30% were 70 years or older in the population of 2137 patients. Almost one-third of patients were > 70 years of age in International Registry of Acute Aortic Dissection (IRAD) [1] and 19.8% were aged 80 years old or older in Japan registry of aortic dissection (JRAD) [8]. Compared with IRAD, the patients with AAD were significantly younger in Chinese registry (Sino-RAD) [9], of which 6.5% were over 70 years old and 25.4% were over 60 years old. Therefore, it can be seen from these worldwide studies that the proportion of elderly patients (> 70 years of age) in China is less than that in western countries. It may be related to the fact that some elderly patients with type A AAD in rural areas die before presentation to hospital, or refuse operation owing to surgical risk and financial problem. In our center, there were only 49 patients aged over 70, accounting for 9.6% in 510 type A AAD patients who underwent surgeries from 2000 to 2015. Because the enrolled sample was too small, we could only expand the age range to ≥65 years old in this study.

Whether surgery is justified in elderly type A AAD patients? Evangelista A and his colleagues [1] clearly compared two groups of below and above 70 years and reported that surgical mortality was 21% in patients < 70 years of age and 31% in those ≥70 years of age. GERAADA [10] concluded age to be a critical risk factor for morbidity and mortality in 1558 type A AAD patients and clearly confirmed the correlation of older age with early mortality in a large study cohort. The finding that identified age ≥ 70 years to be a predictor of death could also be verified in IRAD data [11]. All these literatures would suggest that surgery should be considered in all patients with type A AAD regardless of age; however, these studies also revealed that older age was an independent predictor of surgical mortality.

The controversy that still exists regarding the extent of aortic resection for repair for type A AAD in the general population is even more pertinent in elderly patients. As far as we know, Sun’s procedure (TAR+SET) has acquired huge success in China, which not only repairs of extensive parts of the aorta, including the aortic arch and descending aorta, but also has satisfied surgical outcomes and long-term results with decreased rate of re-operation and low risk of adverse aortic events. Sun and his colleagues [4, 5, 12–14] reported that total arch replacement could be performed safely without increasing operative mortality and morbidity compared with hemi-arch replacement. Studies from abroad [15–19] also reported no significant differences in terms of hospital mortality between hemi-arch replacement and total arch replacement. Another meta-analysis [20] found that no statistical difference was obtained in aspects of neurological deficit, stroke, intubation > 72 h, and re-operation for bleeding except for postoperative renal dialysis between total arch replacement and hemi-arch replacement. But, the outcomes from our current study totally differed from the above previous studies. Here the possible reason could be pointed out is that the average age of patients in above studies was much younger than that in the current study. Therefore, we maintain that the conclusion of total arch replacement carrying the same mortality and morbidity compared with hemi-arch replacement cannot be inferred arbitrarily to elderly patients. Actually in the past decade, the indications for LAR (hemi-arch or ascending aortic replacement) or - TAR+SET for elderly were as following: 1. Basically, we resected the tears in ascending and arch as possible as we could. If no tear in arch or no limb malperfusion, we did LAR. 2. Tear in proximal descending aorta was recommended for TAR+SET operation because this kind tear could be covered by stent elephant trunk. 3. Preoperative limb malperfusion was suggested for TAR+SET operation because stent trunk could expand the true lumen and probably restore the flow to lower extremities. The most important thing we should point out is that we do not hesitate to undergo TAR+SET procedure regardless of age if patients’ condition requires. 4. The exceptional condition was that if one patient was dynamic unstable to tolerate an extensive aorta resection, we would only do LAR to rescue his or her life even if he or she needed extensive surgery. In LAR group, 3 patients with tear locating in arch and 3 locating beyond arch were in these conditions.Therefore, the most important principle is one patient one rule, that is to say, which is made upon individual patient.

Motohiko Goda [21] found that risk factors for hospital mortality in patients with type A AAD were cardiopulmonary resuscitation, renal dysfunction, and lower-extremity ischemia. Another study [22] indicated that pre-existing cardiac disease (RR = 3.7, 95% CI = 1.8–7.4) and cardiopulmonary resuscitation (RR = 6.8, 95% CI = 2.3–20.2) were independent predictors of in-hospital death for 487 type A AAD. Therefore, variables associated with patients’ characteristics and the surgical procedures, such as surgical technique, cannulation site, CPB time, cross-clamp time, and circulatory arrest time with a potential influence on mortality have been investigated a lot in type A AAD. Although most previous studies revealed that hemi-arch and total arch replacement had no significant difference on early death [15–19], it was quite different regarding hospital mortality, which was much higher (19.4%) in TAR+SET group than in LAR group (4.9%) in the current study. This was most likely due to the small number of sample in each operative method.

There is always a fear that patients would die of rupture of the residual false lumen after LAR, although the present study confirmed that elderly patients, who underwent LAR had lower surgical mortality and morbidity than those who underwent TAR+SET in the setting of type A AAD. Residual patent false lumen is a well-known risk factor for progressive aortic dilatation and poor long-term outcomes following type A AAD surgery [16, 23, 24]. Fichadiya A and his colleagues [25] analyzed that false lumen thrombosis was achieved in 57 and 9% of patients undergoing extended-arch (total arch) and hemi-arch repair, respectively. Rate of growth in the proximal descending aorta was 0.7 ± 2.3 mm/year in the extended-arch group versus 2.7 ± 3.9 mm/year in the hemi-arch group. In our present study, expansion of the true lumen and regression of the false lumen was observed in almost all the patients except 3 patients in TAR+SET group. In contrary, for LAR patients, false lumen in aortic arch or proximal descending aorta was observed in 35 discharged patients (35/39, 89.7%) with aortic aneurysm progression during follow-up. The increased diameter of proximal descending aorta was (19.5 ± 9.3) mm, with expansion occurring at (3.1 ± 1.6) mm/y for these 35 patients. The expansion speed is higher than that in Fichadiya A and his colleagues’ study, probably due to poor control of blood pressure in elderly patients in China. Aizawa K and his colleagues [26] compared 225 patients who underwent ascending or hemi-arch replacement and 42 underwent total arch replacement for type A AAD patients and found that the actuarial survival rates were 80.7% for ascending/hemi- arch group versus 84.3% for total arch group after 5 years, and 66.4% for ascending/ hemi-arch group versus 74.6% for total arch group after 10 years (*p* = 0.94). For ascending/hemi-arch and total arch groups, reoperation-free survival rates were 72.1% versus 77.1% after 5 years, and 62.0 versus 67.1%, respectively, after 10 years (*p* = 0.85). For our study, the 5-year survival rate was 82.5 ± 6.0% in LAR group versus 75.2 ± 5.6% in TAR+SET group (*p* = 0.151). But, the freedom from adverse aortic events at 5-year follow-up was (84.3 ± 6.5) % in LAR group versus (97.9 ± 2.1) % in TAR+SET group (*p* = 0.03), which was different from the previous study. We understand the fact that LAR did fail to achieve the objective of massive resection of dissection and realized that average age nearly 70 years old in this study could mainly highlight the shortness of LAR. Despite adverse aortic events risk in LAR group was increased; however, there was no statistically significant difference in mid-term survival rate between the two groups.

There are certain limitations of this study as follows. Firstly, the current study is a retrospective analysis with all known limitations of such a study design. Secondly, the data come from a single center with a small sample; however, it might also decrease the potential bias because surgical, anesthetic and intensive care techniques even the data collection methods are consistent. Thirdly, the choice of procedure is not random, but determined upon single patient’s condition. The difference in aspects of limb malperfusion and tear location exists, which may lead to different procedures. Last one, it is difficult for us to set up a completely consistent control group in clinical. So, we calculated the Euro-score II for the two groups (17.1 ± 5.5 vs. 16.7 ± 6.3, *p* = 0.733), which emphasizes the weight of the patients’ preoperative status.

## Conclusion

The present study revealed that TAR+SET carried a higher mortality and surgical risk than LAR for type A AAD patients aged 65 or older. The 5-year survival rates had no difference between the two groups, although TAR+SET indeed decreased adverse aortic events in follow-up. These findings support limited aortic resection is acceptable for patients aged over 65 with type A AAD if surgical principles allow.

## Data Availability

The datasets used and/or analysed during the current study are available from the corresponding author on reasonable request.

## Abbreviations

AAD: Type A acute aortic dissection
LAR: Limited aortic resection
TAR+SET: Total aortic arch replacement and stent elephant trunk implantation in the descending aorta
TEE: Transesophageal echocardiography
CPB: Cardiopulmonary bypass
SCP: Selected cerebral perfusion
AMI: Acute myocardial infarction
AVR: Aortic valve replacement
AI: Aortic valve insufficiency
CABG: Coronary artery bypass graft
CVA: Cerebrovascular accident
STC: Sinus-tube conjunction
ICU: Intensive care unit
GERAADA: German Registry for Acute Aortic Dissection Type A
IRAD: International Registry of Acute Aortic Dissection
JRAD: Japan registry of aortic dissection
